# Analyzing the Costs and Benefits of Utilizing a Mixed-Strategy Approach in Infectious Disease Control under a Voluntary Vaccination Policy

**DOI:** 10.3390/vaccines11091476

**Published:** 2023-09-11

**Authors:** K. M. Ariful Kabir, Mohammad Sharif Ullah, Jun Tanimoto

**Affiliations:** 1 Department of Mathematics, Bangladesh University of Engineering and Technology, Dhaka 1000, Bangladesh; 2 Department of Mathematics, Feni University, Feni 3900, Bangladesh; msu@feniuniversity.ac.bd; 3 Interdisciplinary Graduate School of Engineering Sciences, Kyushu University, Fukuoka 8168580, Japan; tanimoto@cm.kyushu-u.ac.jp; 4 Faculty of Engineering Sciences, Kyushu University, Fukuoka 8168580, Japan

**Keywords:** cost–benefit, mixed-strategy, vaccination game, repeated season

## Abstract

Infectious diseases pose significant public health risks, necessitating effective control strategies. One such strategy is implementing a voluntary vaccination policy, which grants individuals the autonomy to make their own decisions regarding vaccination. However, exploring different approaches to optimize disease control outcomes is imperative, and involves assessing their associated costs and benefits. This study analyzes the advantages and disadvantages of employing a mixed-strategy approach under a voluntary vaccination policy in infectious disease control. We examine the potential benefits of such an approach by utilizing a vaccination game model that incorporates cost and benefit factors, where lower costs and higher benefits lead to reduced infection rates. Here, we introduce a mixed-strategy framework that combines individual-based risk assessment (IB-RA) and society-based risk assessment (SB-RA) strategies. A novel dynamical equation is proposed that captures the decision-making process of individuals as they choose their strategy based on personal or communal considerations. In addition, we explore the implications of the mixed-strategy approach within the context of social dilemmas. We examine deviations from expected behavior and the concept of social efficiency deficit (SED) by allowing for the evolution of vaccine strategy preferences alongside risk perception. By comprehensively evaluating the financial implications and societal advantages associated with the mixed-strategy approach, decision-makers can allocate resources and implement measures to combat infectious diseases within the framework of a voluntary vaccination policy.

## 1. Introduction

Infectious diseases present serious public health threats, necessitating effective control measures. One such approach is a voluntary vaccination policy, which respects individual decision-making. However, to optimize disease control, exploring diverse strategies and weighing their costs and benefits is vital. This study analyzes the merits and drawbacks of a mixed-strategy approach within such a policy. By combining individual and societal risk assessments, we aim to uncover potential advantages while addressing social dilemmas and efficiency deficits. This research informs decision-makers about resource allocation with regard to combating infectious diseases within voluntary vaccination frameworks.

The application of the evolutionary game [1] theory when analyzing the dynamics of epidemic models has provided valuable insights into the behavior of infectious diseases and the effectiveness of various intervention strategies [2,3,4]. One critical aspect of epidemic control is vaccination [5,6,7], which aims to mitigate the spread of diseases by immunizing individuals. However, vaccination programs [8,9,10,11,12] have associated costs and benefits that must be carefully evaluated to maximize their effectiveness. This paper uses evolutionary game theory to explore the concept of cost–benefit vaccination in the context of an epidemic model. By considering the trade-offs between the costs of vaccination and the benefits of reduced infection rates and disease burden, this study aims to shed light on the optimal strategies for vaccine distribution and allocation.

In epidemic-based evolutionary game theory models, incorporating both individual and society-based risk assessment is crucial to a comprehensive understanding of disease dynamics and the impact of intervention strategies [13,14]. Individual-based risk assessment (IB-RA) involves evaluating the risks and benefits of vaccination for a single individual, considering personal factors such as susceptibility to the disease, potential side effects, and perceived benefits of vaccination. On the other hand, society-based risk assessment (SB-RA) considers the collective welfare of the community, assessing the overall benefits and costs of vaccination at a population level. By integrating these two concepts, one can develop sophisticated models that capture the complex interplay between individual decision-making and community outcomes, providing insights into optimal vaccination strategies and addressing potential dilemmas between personal and societal interests. This approach helps inform policies that promote the common good while respecting individual autonomy.

Epidemic compartmental models [15,16] are instrumental when it comes to comprehending and forecasting the dissemination of contagious diseases among communities. Furthermore, they facilitate the acquisition of insights into the behavior of diseases and inform decision-making processes for effective disease control and prevention strategies. For instance, the SVIR (susceptible–vaccinated–infected–recovered) mathematical epidemiological model is a mathematical framework used to analyze and comprehend the population-wide transmission of infectious diseases [17,18]. Recognizing the impact of vaccination campaigns on disease dynamics, the SVIR model incorporates an extra compartment to account for vaccinated (V) individuals. Susceptible individuals are at risk of contracting the disease, vaccinated individuals have received a vaccine and accomplished protection against infection, infected individuals are actively spreading the disease, and recovered individuals have either survived the infection and developed immunity or have been successfully treated.

Evolutionary game theory (EGT) has emerged as a valuable mathematical framework for studying cooperative behavior in various domains, including epidemics [19,20]. Bauch and Bhattacharyya [21] proposed incorporating EGT into the analysis of individual behavior within an epidemic context. Chen and Fu [22] applied social learning theory to examine decision-making related to vaccination and self-isolation during health crises. Zho et al. [23] studied the impact of determined individuals on voluntary vaccination behavior based on information-driven decisions and benefit–cost analysis. Lim and Zhang [24] investigated factors influencing vaccination choices using a nonlinear public good game. Other studies explored the effects of intermediate defensive mechanisms [25], developed analytical frameworks for vaccination games [26], and examined the influence of individual imitation and population structure on achieving widespread immunity [27]. Kabir et al. [28,29] assessed behavioral incentives in vaccination scenarios and proposed models to guide policymaking. Various other studies employed EGT to analyze economic shutdowns during the COVID-19 pandemic [30], examine the impact of human behavior and memory [31], and study the effects of vaccination and treatment on epidemic transmission patterns [32].

Furthermore, it is worth noting that the beneficial effects of vaccination in the EGT model have yet to be noticed by previous authors. In this study, we aim to fill this gap by incorporating the positive impact of vaccination into the EGT framework. By considering the beneficial effects of vaccination, we can elucidate how it influences human decision-making and, consequently, aid policymakers in implementing effective measures to curb the spread of infectious diseases. Moreover, integrating individual and society-based risk assessment within these sophisticated models allows for a comprehensive understanding of the intricate dynamics between individual choices and community outcomes. This approach provides valuable insights into optimal vaccination strategies while addressing potential conflicts between personal and societal interests.

In recent years, a growing body of research has focused on the dynamics of epidemic control strategies, commonly known as the intervention game, both theoretically and numerically [33,34,35,36,37]. Bauch et al. [21,38] and others [17,32,33,34,37] adopted an approach considering a scenario in which disease spread and individuals’ behavioral changes due to social learning evolve simultaneously within one season, resembling real-world dynamics in specific social contexts. However, Kuga et al. [13] and Kabir et al. [14] developed vaccination epidemic game models, respectively, which consider the spread of the disease within a single season, referred to as the “local time scale,” with strategy updates occurring at the end of each season on the “global time scale” or generation. We also investigated the concept of Social Efficiency Deficit (SED) [39] to understand the social dilemma better. In line with this concept, we aim to construct a mathematical formulation of the vaccination game that accounts for disease transmission and strategy updates for vaccination behavior within different time scales.

## 2. Model Formulation

**Figure 1:**
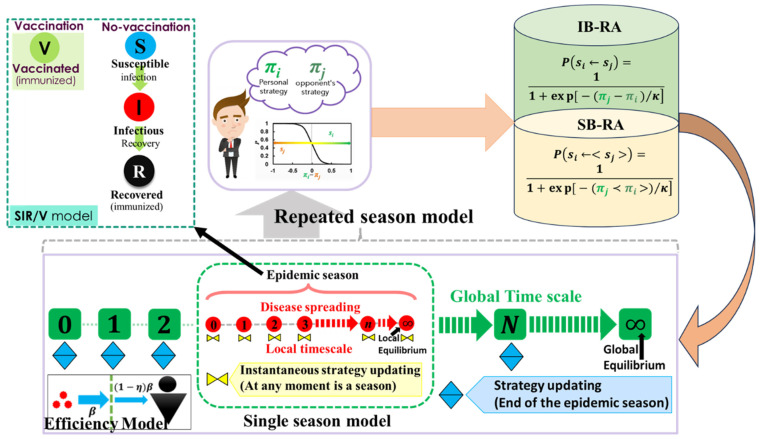
Schematic diagram of the model in which the population is divided into four states: susceptible (S), vaccinated (V), infected (I), and recovered (R), which applies in the epidemic season on a local time scale. On the other hand, the evolutionary decision-making process based on the Fermi pairwise game occurs globally. An individual chooses whether to vaccinate at the onset of each epidemic season based on two updated dynamics: IB-RA (individual-based risk assessment) and SB-RA (society-based risk assessment). The vaccine efficiency (VE) models determine the fraction of vaccinated and corresponding immunity systems.

The efficiency of a defense against contagion to prevent infection should be expressed as η(0≤η≤1), which refers to how the defensive measure can reduce the likelihood of contracting the virus. In constructing the following efficiency model, we consider the vaccinated state as the state that is ready to defend against contagion. Utilizing the compartment model, which allows people in a community to be categorized into susceptible (S), infected (I), recovered (R), and vaccinated (V) stages, we explain the dynamics of a propagating epidemic (see Figure 1). If exposed to infectious persons at the disease transmission rate of β (per day per person), an unvaccinated, susceptible person who is vulnerable to infection (more accurately, one who is unprepared with the defense against contagion) may contract the disease. A person in S compartment equipped with resistance against contagion may also contract the infection at the rate (1−η)β. The rate of recovery for an infected person is γ (per day). Thus, the system of nonlinear differential equations to represent the SIR/V model is

(1a) $$\frac{dS}{dt}=-\beta S\left(x,t\right)I\left(x,t\right),\;\;\;\;$$

(1b) $$\frac{dV}{dt}=-\beta \left(1-\eta \right)V\left(x,t\right)I\left(x,t\right),\;\;\;\;\;$$

(1c) $$\frac{dI}{dt}=\beta S\left(x,t\right)I\left(x,t\right)+\beta \left(1-\eta \right)V\left(x,t\right)I\left(x,t\right)-\gamma I\left(x,t\right),$$

(1d) $$\frac{dR}{dt}=\gamma I\left(x,t\right).\;\;\;\;\;\;$$

Therefore,

(1e) $$S\left(x,t\right)+V\left(x,t\right)+I\left(x,t\right)+R\left(x,t\right)=1.\;$$

The utilization of the control reproduction number $R_{c}$, rather than the basic reproduction number $R_{0}$, is appropriate due to the presence of a non-completely susceptible population. In this particular scenario, the estimation of $R_{c}$ can be determined as:

(2) $$R_{c}=\frac{\beta }{\gamma }\left[S\left(x,0\right)+\left(1-\eta \right)V\left(x,0\right)\right]=R_{0}\left[S\left(x,0\right)+\left(1-\eta \right)V\left(x,0\right)\right]\;\;\;\;\;$$

With the initial conditions: $S\left(x,0\right)=1-x,V\left(x,0\right)=x,$ and $I\left(x,0\right)=0$, the ultimate magnitude of the epidemic and its various proportions can be represented as [13,14],

(3a) $$S\left(x,0\right)=\left(1-x\right)exp\left[-R_{0}R\left(x,\infty \right)\right],\;$$

(3b) $$V\left(x,0\right)=xexp\left[-\left(1-\eta \right)R_{0}R\left(x,\infty \right)\right],$$

(3c) $$R\left(x,0\right)=1-\left(1-x\right)exp\left[-R_{0}R\left(x,\infty \right)\right]-xexp\left[-\left(1-\eta \right)R_{0}R\left(x,\infty \right)\right].$$

At the point of convergence in this process, the proportions of the four distinct categories of individuals at a state of equilibrium are presented concisely in Table 1.

### 2.1. Cost–Benefit Payoff Matrix

The evolutionary process incorporates two distinct decision-making strategy updates: individual-based risk assessment (IB-RA) and society-based risk assessment (SB-RA). According to the prescribed procedure, it is assumed that all rules dictate a synchronized strategy update for each individual after every flu season during repeated vaccination seasons.

In a vaccination program, individuals can participate by paying a cost ($C_{v}$) for the vaccine, which comes with benefits (B); B represents the healthy situation. Let us introduce the cost of infection as $C_{d}$. Generally, *B* does mean much more than a cost gap with regard to receiving a vaccination ($C_{v}$) as well as infection ($C_{d}$). That is because *B* also confers a sort of psychological advantage related to their ability to escape infection. Alternatively, individuals can choose not to get vaccinated, incurring no cost but still reaping the benefits (B) through free riding. If individuals participate but get infected, their payoff is $-C_{v}-C_{d}$, while failed free riders (unvaccinated and infected) have a payoff of $-C_{d}$. Four distinct groups emerge: those who are vaccinated and healthy (HV), vaccinated but infected (IV), successful free riders (SFR) who remain unvaccinated but healthy, and failed free riders (FFR). These groups represent the various outcomes individuals experience based on their vaccination decisions and infection statuses (Table 2).

Without loss of generality, to maintain the general applicability, we can introduce an “additive” operation in Table 2, as PW-Fermi focuses solely on the payoff gap. Therefore, by subtracting the value of *B* from all elements, we obtain Table 3.

By normalizing Table 3 with regard to the sum of $C_{d}$ and $C_{d}$ as ($C_{d}+B$), we obtain Table 4, as shown below.

By introducing another normalized parameter, $\frac{C_{v}}{C_{d}+B}\equiv C_{r}$, it is possible to achieve the exact same payoff structure as shown in Table 5, which is the original one. This means that the conventional vaccination game [13] encompasses the proposed model (Table 6). In conclusion, through appropriate normalization and the inclusion of $C_{r}$ (relative cost of vaccination), the proposed model aligns with the standard framework of the vaccination game, highlighting its compatibility and consistency with existing models in the field.

Now, the expected payoffs can be assessed by calculating the average social payoff <π>, the average corporative (vaccinated) payoff ${<\pi }_{C}>$, and the average defective (non-vaccinated) payoff ${<\pi }_{D}>$ for the respective provisions, namely imperfect vaccination and defense against contagion.

(4) $$<\pi >=-\frac{C_{v}}{C_{d}+B}\left(x\;exp\left[-\left(1-\eta \right)R_{0}R\left(x,\infty \right)\right]\right)+\left(-\frac{C_{v}}{C_{d}+B}-1\right)(x(1-exp\left[-\left(1-\eta \right)R_{0}R\left(x,\infty \right)\right])+\left(-1\right)(1-x)(1-exp\left[-R_{0}R\left(x,\infty \right)\right]),$$

(5) $${<\pi }_{C}>=-\frac{C_{v}}{C_{d}+B}\left(x\;exp\left[-\left(1-\eta \right)R_{0}R\left(x,\infty \right)\right]\right)+\left(-\frac{C_{v}}{C_{d}+B}-1\right)(x\left(1-exp\left[-\left(1-\eta \right)R_{0}R\left(x,\infty \right)\right]\right)$$

(6) $$<\pi_{D}>=\left(-1\right)\left(1-x\right)\left(1-exp\left[-R_{0}R\left(x,\infty \right)\right]\right)\;$$

### 2.2. Evolutionary Dynamical Equation

Updating strategies is an effective practice after each epidemic flu season, as previously defined. The mean-field approximation is utilized to model the potential increase or decrease in the vaccinator fraction, denoted as x. Therefore, the following two categories of evolutionary dynamics are being taken into account:

### 2.3. IB-RA (Individual-Based Risk Assessment) 

The cognitive process of an actor considering the option of relying on a single neighbor in an updating manner is called individual-based risk assessment (IB-RA) [13,14]. Assuming that “i” and “j” represent the respective payoffs of an individual actor and their neighbor, the probability in this scenario is denoted as $Pr\left(s_{i}\leftarrow s_{j}\right)\;$and expressed using the Fermi pairwise function as outlined in Table 7. The dynamical equation is as follows:

$${\dot{x}}_{IB-RA}=HV(x,\;\infty )SFR(x,\;\infty )[P_{SFR\leftarrow HV}-P_{HV\leftarrow SFR}]$$

$$\;\;+HV(x,\;\infty )FFR(x,\;\infty )[P_{FFR\leftarrow HV}-P_{FFR\leftarrow HV}]$$

$$+IV(x,\;\infty )SFR(x,\;\infty )[P_{SFR\leftarrow IV}-P_{IV\leftarrow SFR}]\;$$

(7) $$+IV(x,\;\infty )FFR(x,\;\infty )[P_{FFR\leftarrow IV}-P_{IV\leftarrow FFR}].$$

### 2.4. SB-RA (Society-Based Risk Assessment) 

Within the context of societal risk assessment, an individual actor can evaluate their potential gain against that of a collective group or the broader society [13,14]. The modified Fermi pairwise function articulates the mean anticipated gain obtained by averaging the proportion of the similar tactic, denoted as $Pr\left(s_{i}\leftarrow {<\pi }_{j}>\right),$ as presented in Table 8. The dynamical equation is as follows:

(8) $${\dot{x}}_{SB-RA}=HV\left(x,\;\infty \right)NV\left(x,\;\infty \right)P_{HV\leftarrow NV}-IV\left(x,\;\infty \right)NV\left(x,\;\infty \right)P_{IV\leftarrow NV}+SFR\left(x,\;\infty \right)V\left(x,\;\infty \right)P_{SFR\leftarrow V}\;+FFR\left(x,\;\infty \right)V\left(x,\;\infty \right)P_{FFR\leftarrow V}\;\;\;\;\;\;\;\;\;\;\;\;\;\;\;\;\;\;\;\;\;\;\;\;$$

### 2.5. Mutual Strategy Selection Dynamics

In our approach, we utilize a combined strategy selection method that incorporates two strategies: IB-RA and SB-RA. Whether an individual chooses to get vaccinated is determined by a parameter called selection intensity, denoted as n. When n is set to zero or one, individuals switch their strategy based solely on IB-RA or SB-RA, respectively. However, for values of n between 0 and 1, individuals modify their strategy using a combination of IB-RA and SB-RA update rules. Let x be defined as follows:

(9) $$\dot{x}=(1-n)\cdot {\dot{x}}_{IB-RA}+n\cdot {\dot{x}}_{SB-RA}$$

The SB-RA strategy is considered the primary method for updating procedures, in which individuals compare their strategy with that of the society or community. Individuals tend to prefer updating their approach based on the choices of their immediate neighbors. However, as the proportion of cooperators (vaccinators) in the population increases, individuals who wish to update their strategy will increasingly look at the overall community rather than just their immediate neighbors. Hence, we can define the dynamics of individual selection as follows:

(10) $$\dot{n}=\omega n\left(1-n\right)\left[\;\theta x-\left(1-x\right)\right].\;$$

The social behavior within the society is influenced by the scalar value n(1−n), where the term n(1−n) ensures that the state of the community remains within the range of [0,1]. Additionally, the parameter θ, referred to as the enhancing parameter, signifies the relative speed at which the cognitive behavior in society transforms compared to the frequency of rational actors (strategists or individuals making strategic vaccination decisions).

The Social Efficiency Deficit [40] captures the extent to which the evolutionary equilibrium falls short of the social optimum. It measures the potential inefficiency or suboptimality arising from self-interested decision-making without considering the broader societal welfare. By quantifying this disparity, we can better understand the implications of individual actions and the potential gains that could be achieved by moving closer to the social optimum. Thus, the Social Efficiency Difference (SED) is a metric that can be used to assess the gap between the expected benefits in an ideal social optimum scenario and the outcomes that emerge naturally through evolutionary processes. The Social Efficiency Difference (SED) refers to the difference between the expected overall benefits in a social optimum scenario ($\Pi_{SO}$) and those in an evolutionary equilibrium situation ($\Pi_{NE}$).

Therefore,

(11) $$SED=\Pi_{SO}-\Pi_{NE}.$$

The deviation of the final epidemic size refers to the disparity between the values of the final epidemic size at the social optimum and those at the evolutionary equilibrium. It quantifies the difference in the size of the epidemic when comparing the ideal scenario in which societal welfare is maximized to the outcome resulting from self-interested decision-making. Similarly, the deviation of the fraction of vaccination refers to the difference between the values of the fraction of individuals vaccinated at the social optimum and those at the evolutionary equilibrium. It quantifies the discrepancy in vaccination rates between the ideal scenario in which societal welfare is maximized and the outcome resulting from self-interested decision-making. Therefore, the deviation of the final epidemic size and the deviation of the vaccination fraction provide measures of the disparities between the optimal outcomes at the social optimum and those resulting from self-interested decision-making. These deviations help us assess the suboptimality of individual decision-making and identify potential areas for improvement to achieve better epidemic control and vaccination rates.

Therefore,

(12a) $$D_{FES}={FES}^{SO}-{FES}^{NE}$$

(12b) $$D_{FOV}={FOV}^{SO}-{FOV}^{NE}\;\;\;.$$

## 3. Result and Discussion

**Figure 2:**
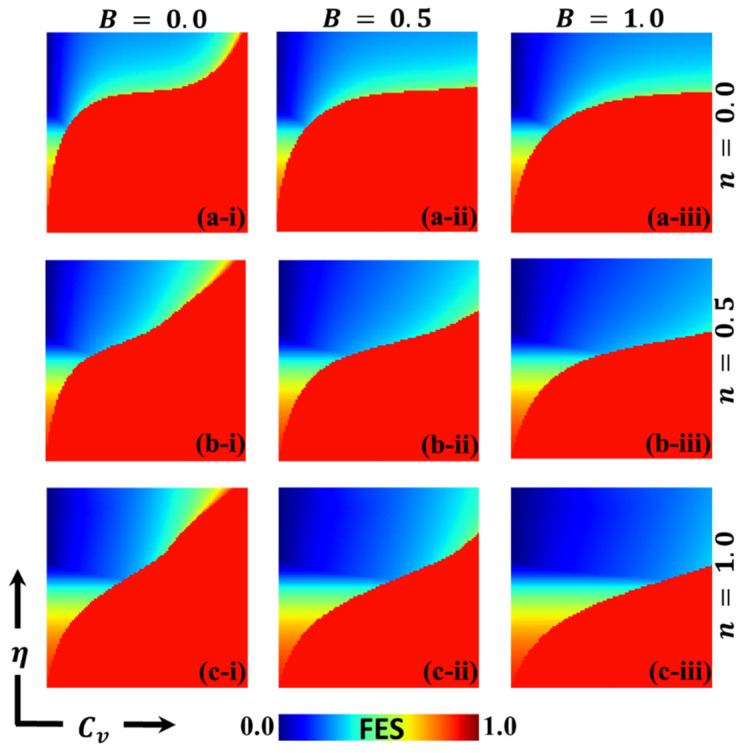
The 2D heatmap of the final epidemic size (FES) is presented by varying two parameters: the x-axis contains the vaccination cost ($C_{v}$), and the y-axis shows the vaccination efficacy (η). In this figure, the first, second, and third rows display the result of varying the selection intensity rate (a-*) *n* = 0.0, (b-*) *n* = 0.5, and (c-*) *n* = 1.0. Also, the first, second, and third columns show the result of varying the benefit rate: (*-i) B=0.0, (*-ii) B=0.5, and (*-iii) B=1.0. Other parameters are β=0.8333, γ=0.333 [13,14].

**Figure 3:**
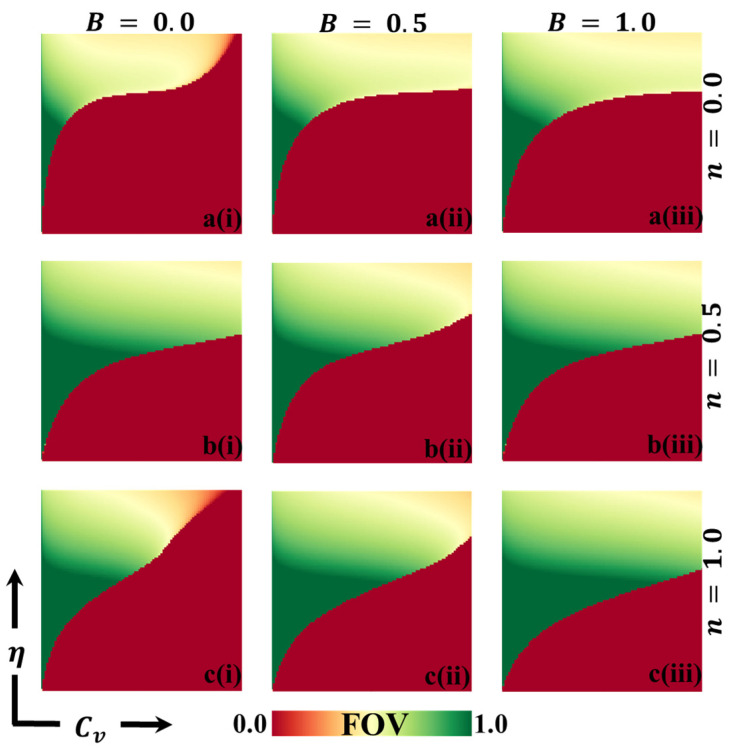
The 2D heatmap representing the fraction of vaccination (FOV) illustrates variations in two parameters: the x-axis represents the vaccination cost ($C_{v}$), while the y-axis represents vaccination efficacy (η). In this figure, we present results for different combinations of selection intensity rates—specifically, (a-*) n=0.0 in the first row, (b-*) n=0.5 in the second row, and (c-*) n=1.0 in the third row. Additionally, the first, second, and third columns depict results for different benefit rates: (*-i) B=0.0, (*-ii) B=0.5, and (*-iii) B=1.0. It is important to note that we have kept other parameters constant, with β=0.8333 and γ=0.333 [13,14].

**Figure 4:**
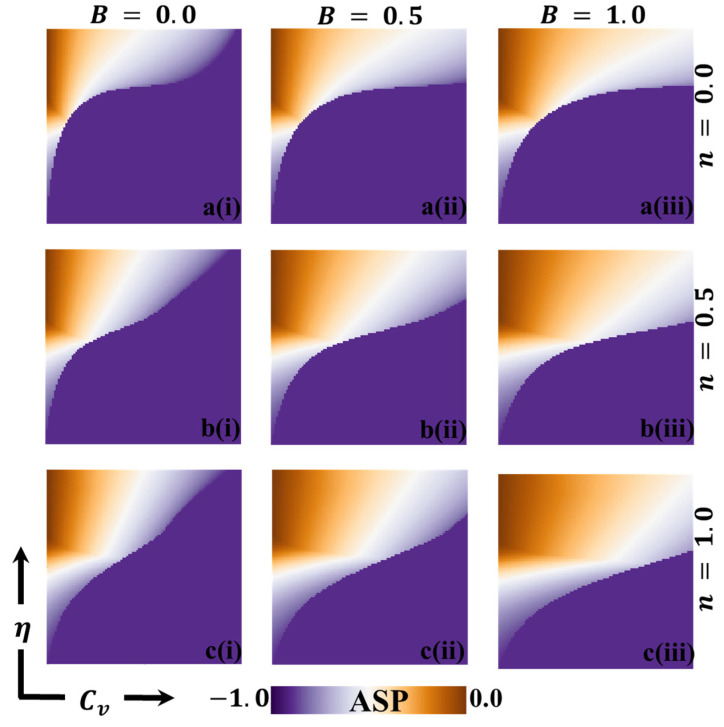
The 2D heatmap, which represents the average social payoff (ASP), demonstrates variations in two key parameters: the x-axis signifies the vaccination cost ($C_{v}$), while the y-axis represents vaccination efficacy (η). In this visual representation, we showcase outcomes for various combinations of selection intensity rates, denoted as n, with (a-*) n=0.0 in the first row, (b-*) n=0.5 in the second row, and (c-*) n=1.0 in the third row. Furthermore, the first, second, and third columns display results for different benefit rates, identified as (*-i) B=0.0, (*-ii) B=0.5, and (*-iii) B=1.0, respectively. It is important to emphasize that we have maintained the stability of other parameters throughout, with β=0.8333 and γ=0.333 [13,14].

**Figure 5:**
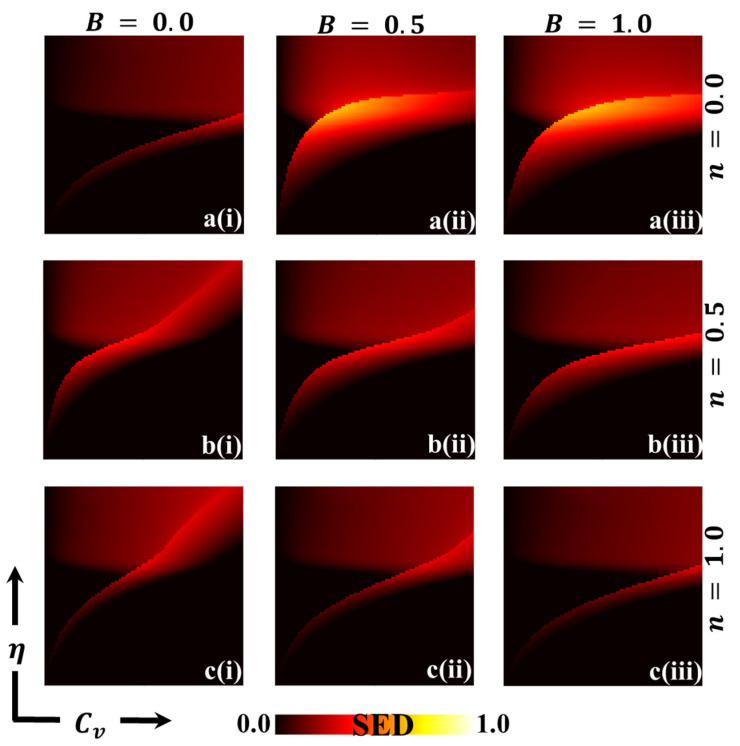
The 2D heatmap depicting the social efficiency deficit (SED) is generated by manipulating two key parameters: the x-axis represents the vaccination cost ($C_{v}$), while the y-axis signifies vaccination efficacy (η). Within this graphical representation, the results are organized into three rows, each presenting variation in the selection intensity rate n: the first row corresponds to (a-*) n=0.0, the second to (b-*) n=0.5, and the third to (c-*) n=1.0. Similarly, the results are arranged into three columns, each reflecting change in the benefit rate: (*-i) B=0.0 in the first column, (*-ii) B=0.5 in the second column, and (*-iii) B=1.0 in the third column. We must note that we have maintained the constancy of other parameters throughout, with β=0.8333 and γ=0.333 [13,14].

**Figure 6:**
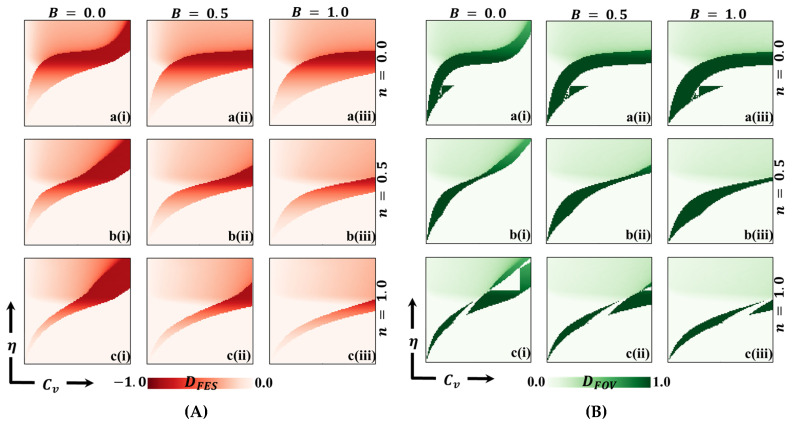
We present a 2D heatmap illustrating two aspects: (**A**) the deficiency of final epidemic size ($D_{FES}$) and (**B**) the deficiency of the fraction of vaccination ($D_{FOV}$). These visualizations involve the manipulation of two key parameters: the x-axis represents the vaccination cost ($C_{v}$), while the y-axis represents vaccination efficacy (η). Within this figure, you will find three rows, each showcasing the outcomes of varying the selection intensity rate (a-*) with values of n=0.0 in the first row, n=0.5 in the second row, and n=1.0 in the third row. Additionally, the figure features three columns, each presenting the results of varying the benefit rate: (*-i) B=0.0 in the first column, (*-ii) B=0.5 in the second column, and (*-iii) B=1.0 in the third column. It is important to note that we have kept other parameters constant throughout the analysis, specifically β=0.8333 and γ=0.333 [13,14].

**Figure 7:**
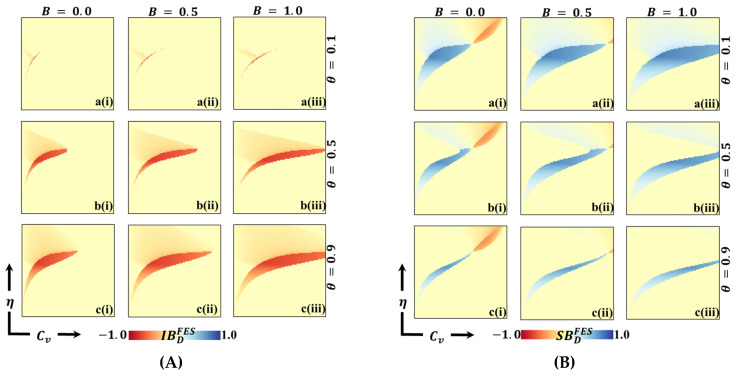
We present a 2D heatmap representing (**A**) individual-based deficiency of final epidemic size $\left({IB}_{D}^{FES}\right)$ and (**B**) society-based deficiency of fraction of vaccination $\left({SB}_{D}^{FES}\right)$. These visualizations involve the manipulation of two key parameters: the x-axis denotes the vaccination cost ($C_{V}$), while the y-axis represents vaccination efficacy (η). Within this figure, you will find three rows, each showcasing the outcomes of varying the process parameter (a-*) with values of θ=0.1 in the first row, θ=0.5 in the second row, and θ=0.9 in the third row. Additionally, the figure features three columns, each presenting the results of varying the benefit rate: (*-i) B=0.0 in the first column, (*-ii) B=0.5 in the second column, and (*-iii) B=1.0 in the third column. It is important to note that we have maintained the constancy of other parameters throughout the analysis, specifically β=0.8333 and γ=0.333 [13,14].

In this discussion, we examine the impact of vaccination on reducing disease transmission and analyze the cost–benefit effects of a vaccination game. To gain a deeper understanding, we assign values to various variables for evaluation purposes. These variables include the final epidemic size (FES), the fraction of vaccination coverage (FOV), the average social payoff (ASP), the social efficiency deficit (SED), the deviation of FES $\left(D_{FES}\right)$, and the deviation of vaccination coverage $\left(D_{VC}\right)$ at each social equilibrium. We compare the outcomes using two different strategy update rules: individual-based risk assessment (IB-RA) and society-based risk assessment (SB-RA). The comparison is presented in a 2D phase diagram illustrating the relationship between the vaccination cost $\left(C_{v}\right)$ and vaccination efficiency (η). Figure 2, Figure 3, Figure 4, Figure 5, Figure 6 and Figure 7 showcase the outcomes of the individual-based risk assessment (IB-RA), society-based risk assessment (SB-RA), and a combination of both strategies (intermediate) for three distinct levels of strategy selection intensity rate, (a-*) n=0.0, (c-*) n=1.0, and (b-*) n=0.5, respectively. The figures also depict three different values of benefit rate (*-i) B=0.0, (*-ii) B=0.5*,* and (*-iii) B=1.0, which are utilized to adjust the vaccine cost and efficiencies.

The findings from Figure 2 (*-i) with a benefit rate of B=0.0 (without any benefits) indicate that during a pandemic, the majority of individuals opt for vaccination either through individual-based risk assessment (IB-RA) when n=0.0 or society-based risk assessment (SB-RA) when n=1.0. This observation aligns with the findings of a previous study [13]. However, individuals who adopt an intermediate strategy (0<n<1), instead of prioritizing either IB-RA or SB-RA, follow an embedded approach to update their strategy regarding the transmission of the infection.

In panel 2(a–i), when the cost of vaccination is higher and its efficiency is negligible, a full-scale spread of the infection becomes inevitable. This is because individuals who are having doubts about its reliability and affordability tend to avoid vaccination altogether. To control the spread of the epidemic, the boundary between the monotone region and the remaining region plays a crucial role in transitioning the phase from a pandemic to a controlled (disease-free) state. In the controlled phase, a distinct blue area signifies lower infection rates due to higher efficiency and lower cost. Similarly, detailed full-phase diagrams for panels 2(b–i) and 2(c–i), representing the intermediate strategy and SB-RA approach, respectively, exhibit differences. However, the overall trend remains similar to that which is observed in Figure 2(a–i) in certain aspects. Increasing the value of n (strategy selection intensity rate) reduces the size of the red region and expands the disease-free (blue) region. The updating rule based on global knowledge (SB-RA) rather than local knowledge (IB-RA) provides a more effective means of suppressing the spread of the disease.

When we shift our attention to the benefit parameter B in panels 2(*-ii) and 2(*-iii), we notice that as B increases, the disease-free region, represented by the blue region, expands, meaning that a more significant portion of the population remains unaffected by the epidemic. Additionally, an increase in B leads to a decrease in the final epidemic size, which refers to the total number of individuals affected by the disease. In other words, by increasing the benefit parameter B, we enhance the efficacy of measures or interventions to prevent the spread of the disease. This could involve various actions such as public health campaigns, vaccination efforts, improved healthcare infrastructure, and other strategies designed to limit transmission and mitigate the epidemic’s impact.

Figure 3 illustrates the results obtained by assuming different information benefit rates (B) concerning a fraction of vaccinators (FOV). The depicted scenarios correspond to B values of 0.0, 0.5, and 1.0. Additionally, three types of strategy-updating aspects are shown: (a-*) IB-RA, (b-*) intermediate, and (c-*) SB-RA. These aspects are associated with varying vaccination costs and efficiencies. In the controlled epidemic phase, we can observe a green-colored region in the diagrams, indicating a high vaccination coverage. A combination of higher efficiency and lower vaccination prices characterizes this region. It is interesting to note that even when a significant portion of the population receives the vaccination, the epidemic cannot be eliminated due to the lower reliability of the vaccine. Figure 4 likely provides additional information about the average social payoff (ASP). Although they are different from Figure 4, the detailed full-phase diagrams follow a similar overall trend. Overall, the findings from Figure 3 and Figure 4 suggest that the information benefit rates (B), along with the selection of strategy updating aspects and associated vaccination costs and efficiencies, play crucial roles in shaping the outcomes of the epidemic control efforts. Higher B values and effective strategies can increase vaccination coverage and mitigate the epidemic’s impact. However, despite high vaccination rates, the reliability of the vaccine remains a critical factor in determining the success of epidemic elimination.

Figure 5 presents a 2D heat map illustrating the social efficiency deficit (SED) as it varies, with $C_{v}$ and η represented. We consider every combination of $C_{v}$ and η to calculate the Average Social Payoff (ASP) at the social equilibrium (NE). Additionally, we estimate the ASP at the social optimum (SO) without employing the game approach by determining the maximum ASP for each $C_{v}$ and η while varying the vaccination coverage (x) from 0 to 1. We then calculate the difference between the ASP at SO and NE using a defined Equation (11), which gives us the SED value. In the heat map, the black region represents no SED and, therefore, no dilemma. In this case, lower efficacy does not motivate people to vaccinate, resulting in a dominant defection (D) state as the social optimum (SO), meaning everyone chooses not to commit to vaccination. Similarly, in the fraction of vaccination coverage (FOV) heat map (Figure 3), the corresponding triangular region displays a dominant defection (D) NE, resulting in identical ASP values (Figure 4) as observed at SO. In other words, the payoff at NE cannot be improved further, leading to the absence of any social dilemma. However, another region characterized by low cost in Figure 5(*-i) exhibits no SED. In the FOV phase diagram (Figure 3), the equivalent area indicates dominant cooperation (C) NE, where all individuals choose to commit to vaccination, despite the efficiency not being very high. Furthermore, the ASPs associated with this region (Figure 4) are nearly identical at NE and SO, indicating the absence of SED and, therefore, no social dilemma occurs. On the other hand, the remaining part of Figure 5(*-i) shows varying SED levels, indicating a social dilemma. In this region, we observe non-monotonic changes in SED when the vaccination cost is not excessively high.

An interesting observation can be made when the benefit parameter is increased, as depicted in Figure 5(*-ii) and 5(*-iii); the IB-RA and SB-RA strategies show contrasting tendencies. In the IB-RA strategy, as the benefit parameter B increases, a non-zero SED becomes more pronounced, indicating that a dilemma arises, implying that there is still room to reduce infection despite trade-offs. This suggests that increasing the benefit parameter in the IB-RA strategy leads to a higher likelihood of encountering a social dilemma. However, the SED is comparatively lower in the SB-RA strategy when the benefit parameter B is higher. This suggests that as B increases, the occurrence of a social dilemma diminishes in the SB-RA strategy. This observation implies that the SB-RA strategy, which involves individual group decisions based on social benefits, is more authentic and beneficial for everyone involved. Therefore, when the benefit parameter is increased, the IB-RA strategy shows a higher presence of the social dilemma, indicating trade-offs and the need for further efforts to reduce infection. However, the SB-RA strategy exhibits lower SED levels with higher values of B, indicating a reduced presence of the social dilemma. The SB-RA strategy emphasizes individual group decisions and social benefits and is considered more advantageous and authentic when it comes to managing the epidemic.

Overall, the heat map in Figure 5 helps visualize the distribution of SED levels across different values of $C_{v},\;\eta$ and n. The absence of SEDs in certain regions reflects scenarios where there is no social dilemma. At the same time, SED in other areas indicates situations in which trade-offs and non-monotonic changes occur in the decision-making process regarding vaccination strategies.

From an in-depth perspective, Figure 6 provides a detailed analysis of the deficiencies of FES and FOV in the 2D heatmap, illustrating the relationship between vaccine efficiency and vaccination cost. Panel A represents the deficiency of FES, while Panel B shows the deficiency of FOV denoted by $D_{FES}$ and $D_{FOV}$, respectively. To calculate the FES and FOV at NE, we consider every combination of $C_{v}$ and η and determine their values. Next, we estimate the FES and FOV at the social optimum (SO) to maximize the overall societal welfare, varying the vaccination coverage (x) from 0 to 1. This allows us to determine the maximality of ASP for different $C_{v}$ and η values at SO. Finally, we obtain the values of $\left(D_{FES}\right)$ and $\left(D_{FOV}\right)$ by taking the difference between the FES/FOV values at SO and NE. These values indicate the extent of deficiency or improvement in FES and FOV when moving from NE to SO.

The Social Efficiency Deficit (SED) solely informs us about the presence or absence of a dilemma situation. In contrast, FES ($D_{FES}$) and FOV ($D_{FOV}$) deficiencies offer actionable insights to address this dilemma. When aiming to reduce the spread of disease, two main approaches can be considered: reducing FES, indicated by the red portion in the figure, to improve the overall SED; or implementing vaccination strategies to mitigate the SED. However, it is essential to acknowledge that FES is, to some extent, influenced by FOV. Increasing vaccination coverage (FOV) generally leads to a decrease in FES. Nevertheless, factors such as free riding and the effects of herd immunity can introduce unexpected dynamics that complicate the relationship between FOV and FES. Comparing Panel A ($D_{FES}$) and Panel B ($D_{FOV}$), it becomes evident that the $D_{FES}$ region is more extensive and prominent than the corresponding the $D_{FOV}$ region. This observation implies that relying solely on implementing vaccination measures may not be sufficient to eradicate infections or resolve the dilemma entirely. It indicates that additional strategies or interventions beyond vaccination are required to effectively mitigate the spread of the disease and alleviate the dilemma. By studying Figure 6, we gain valuable insights that complement the findings from Figure 5 (SED). These insights suggest possible strategies and courses of action that may reduce infection rates while effectively addressing the underlying dilemma. Therefore, Figure 6 serves as a crucial tool for guiding decision-making processes and identifying appropriate measures to combat the spread of the disease and tackle the challenges posed by the social equilibrium dilemma.

**Figure 8:**
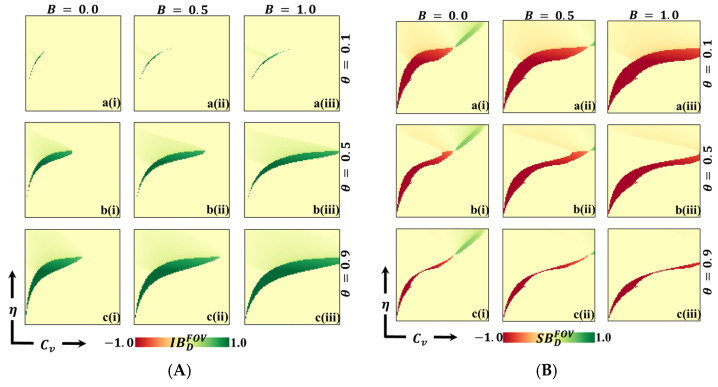
We present a 2D heatmap that illustrates (**A**) the deficiency of vaccination fraction at the individual level $\left({IB}_{D}^{FOV}\right)$ and (**B**) the deficiency of vaccination fraction at the societal level $\left({SB}_{D}^{FOV}\right)$. These visualizations involve the manipulation of two essential parameters: the x-axis corresponds to the vaccination cost ($C_{v}$), and the y-axis represents vaccination efficacy (η). Within this graphical representation, you will find three rows showcasing the outcomes of varying the process parameter (a-*) with values of θ=0.1 in the first row, θ=0.5 in the second row, and θ=0.9 in the third row. Additionally, the figure includes three columns, each displaying the results of varying the benefit rate: (*-i) B=0.0 in the first column, (*-ii) B=0.5 in the second column, and (*-iii) B=1.0 in the third column. It is essential to emphasize that we have maintained the constancy of other parameters throughout the analysis, specifically β=0.8333 and γ=0.333 [13,14].

**Figure 9:**
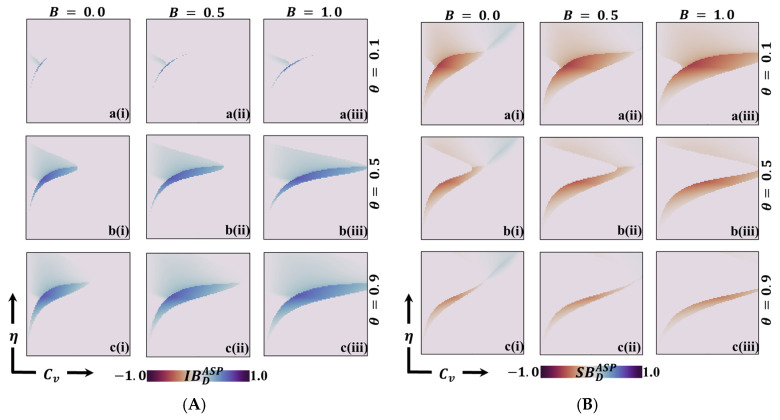
We present a 2D heatmap illustrating (**A**) the individual-based average social payoff $\left({IB}_{D}^{ASP}\right)$ and (**B**) the society-based average social payoff $\left({SB}_{D}^{ASP}\right)$. These visualizations involve the variation of two key parameters: the x-axis represents the vaccination cost ($C_{V}$), and the y-axis denotes vaccination efficacy (η). The first, second, and third rows in this figure delineate the results of altering the process parameter (a-*) with values of θ=0.1 in the first row, θ=0.5 in the second row, and θ=0.9 in the third row. Similarly, the first, second, and third columns portray the outcomes of adjusting the benefit rate: (*-i) B=0.0 in the first column, (*-ii) B=0.5 in the second column, and (*-iii) B=1.0 in the third column. Notably, we have maintained the constancy of other parameters throughout the analysis, specifically β=0.8333 and γ=0.333 [13,14].

Figure 7, Figure 8 and Figure 9 depict 2D graphs illustrating the relationship between $C_{v}$ and η for two scenarios: (Panel A) deviation from Individual-Based Risk Assessment (IB-RA), denoted as $IB_{D}$, and (Panel B) deviation from Society-Based Risk Assessment (SB-RA), denoted as ${SB}_{D}$. These figures provide insights into decision-making outcomes and the effects of changing strategies. To calculate the FES, FOV, and ASP for $IB_{D}$ and $SB_{D}$, we systematically consider every combination of $C_{v}$ and η and determine IB-RA (n=0) and SB-RA (n=1) values. In doing so, we evaluate the baseline outcomes for these two decision-making approaches. Subsequently, we estimate the FES, FOV, and ASP for the dynamic equation of n (as defined in Equation (8)). This allows us to examine decision-making outcomes for different values of theta and assess the influence of dynamic decision-making on these measures. Finally, we calculate the values of $IB_{D}$ and ${SB}_{D}$ by comparing the FES, FOV, and ASP values obtained for the constant values of n (=0 or=1) with those obtained for the dynamic of n. These calculated values highlight how decisions change from myopic or individual-based associations (n=0) to more community-based associations (n=1). These calculations demonstrate the impact of different decision-making approaches on FES, FOV, and ASP, shedding light on how myopic or community-based decision-making strategies influence individual choices and outcomes.

Panels A of Figure 7 and Figure 8 show negative values for ${IB}_{D}^{FES}$ (red) and positive values for ${IB}_{D}^{FOV}$ (green) along the boundary between the disease-free equilibrium and the endemic equilibrium. The negative ${IB}_{D}^{FES}$ indicates that relying on dynamic decision-making (using dynamic n) rather than Individual-Based Rationality (IB-RA) reduces the disease. This observation is also evident in the positive values of ${IB}_{D}^{FOV}$, where disease reduction is observed when entirely positive values occur. Furthermore, as the values of theta increase, the red region (${IB}_{D}^{FES}$) in Figure 7 and the green region (${IB}_{D}^{FOV}$) in Figure 8 expand, implying that as cooperation intensity increases, individuals are more inclined to adopt a community-based decision strategy to reduce the risk of infection. Similarly, increased benefits also lead to a similar tendency towards a community-based decision strategy. Suppose we shift our focus to the Average Social Payoff (ASP). In that case, we can observe a similar trend in FES and FOV, referring to the dynamics of decision making and the resulting outcomes regarding disease reduction align with the patterns observed in FES and FOV. Thus, adopting a dynamic decision-making approach (using dynamic n) instead of relying solely on IB-RA can reduce disease, increasing cooperation intensity, and these benefits further promote adopting community-based decision strategies.

Now, Panel B in Figure 7 and Figure 8 reveals both negative (red) and positive (blue) values for ${SB}_{D}^{FES}$ (deviation from SB-RA) along the boundary region between the disease-free and endemic equilibrium. Notably, in regions characterized by low vaccine cost and low η values, positive ${SB}_{D}^{FES}$ (negative ${SB}_{D}^{FOV}$) is observed, indicating a preference for the SB-RA strategy. Conversely, in regions characterized by higher cost and higher η values, negative ${SB}_{D}^{FES}$ (positive ${SB}_{D}^{FOV}$) is present, indicating a stronger inclination towards relying on the dynamic decision parameter, n. This observed tendency can be attributed to the interplay between higher cost and higher reliability of vaccination. Individuals are more inclined towards vaccination in regions with higher costs and excellent vaccination reliability. However, the higher cost presents a dilemma for individuals, prompting them to seek the benefits of free-riding through the attainment of herd immunity. Consequently, individuals are more prone to relying on the dynamic decision parameter, n, in these circumstances. Interestingly, as the benefit (B) increases (sub-panels (*-ii) and (*-iii)), the red region that represents negative ${SB}_{D}^{FES}$ diminishes. This signifies that increased benefits can help alleviate the dilemma and reduce infection rates by encouraging individuals to participate in vaccination programs. In other words, higher benefits create more substantial incentives for individuals to overcome the dilemma and choose vaccination as a preventive measure.

**Figure 10:**
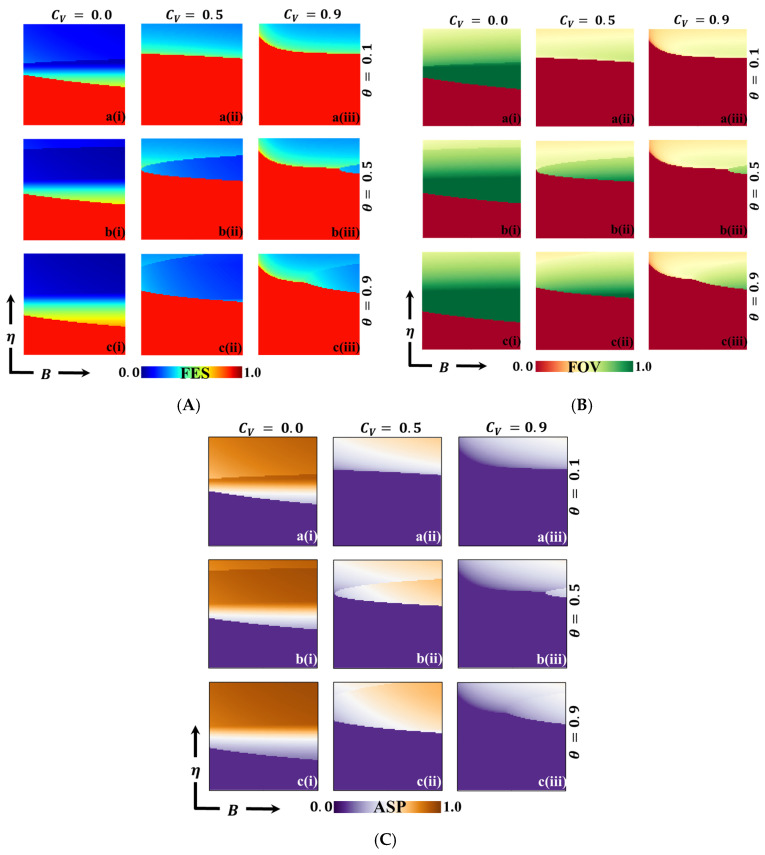
We present a 2D heatmap displaying (**A**) the final epidemic size (FES), (**B**) the fraction of vaccination (FOV), and (**C**) the average social payoff (ASP) while varying two critical parameters: the x-axis represents the vaccination benefit (B) and the y-axis signifies vaccination efficacy (η). Within this graphical representation, the first, second, and third rows showcase the outcomes of altering the process parameter (a-*), with values of θ=0.1 in the first row, θ=0.5 in the second row, and θ=0.9 in the third row. Correspondingly, the first, second, and third columns reveal the results of modifying the vaccination cost: (*-i) $C_{v}=0.1$ in the first column, (*-ii) $C_{v}=0.5$ in the second column, and (*-iii) $C_{v}=0.9$ in the third column. It is noteworthy that we have kept other parameters constant throughout the analysis, specifically β=0.8333 and γ=0.333 [13,14].

As a final step, Figure 10 provides a comprehensive 2D phase-plane analysis, exploring the impact of varying η (vaccine efficiency) and benefit B across relative vaccination costs $C_{v}$. Sub-panels (a-*), (b-*), and (c-*) correspond to different values of θ (=0.1, 0.5, and 0.9, respectively). Across all cases, it is evident that vaccine efficiency (η) influences both FES and FOV, with higher η values leading to a reduction in FES. This outcome aligns with expectations, as higher vaccination reliability makes individuals more inclined to take vaccines. Additionally, as both η and benefit B increase, FES decreases significantly. This result is intuitive, as individuals are more likely to participate in vaccination programs when both the reliability of the vaccine and the associated benefits are high. Consequently, a substantial reduction in FES is observed. Furthermore, the relative vaccination cost ($C_{r}$) is crucial in reducing infection rates, but only when the vaccine cost is relatively low. In such cases, the impact of vaccination costs on infection reduction is significant. Interestingly, as θ (the updating process parameter) increases, a paraboloid-shaped region emerges in the vaccination (and FES) region. This is accompanied by an increase in FOV and a reduction in FES. The presence of the paraboloid-shaped region suggests the occurrence of the non-free-riding effect, mainly when vaccine efficiency is relatively intermediate. These findings underscore the complementary relationship between vaccine benefits, individual strategy selection processes, and their impact on disease control. Policymakers can consider the costs and benefits associated with participation in vaccination programs. However, the selection of individual strategies is determined by the nature of the updating process. This indicates the importance of understanding the decision-making dynamics and designing effective strategies to encourage vaccine uptake and mitigate disease spread. The results also highlight the role of these factors in shaping FES and FOV while revealing the effects of the updating process and the presence of free-riding dynamics. These insights can inform policymakers in devising effective strategies to promote vaccine participation and combat the spread of infectious diseases.

## 4. Conclusions

The ongoing pandemic has highlighted the crucial role of human behavior in responding to and controlling diseases. Evolutionary game theory provides a valuable framework for studying the dynamics of human behavior and how it influences intervention strategies in natural systems. To address this, we have developed an evolutionary game theoretical approach that considers vaccination’s benefits and relative costs, incorporating a global time-scale analysis of epidemic disease dynamics. Our study integrated two strategy-updating processes, namely Individual-Based Random Aspiration (IB-RA) and Social-Based Random Aspiration (SB-RA), into the evolutionary game theory framework. In doing so, we explored how different dynamics and factors impact the outcomes of epidemics, mainly when the relative cost of vaccination is lower, and the benefits derived from vaccination are higher. This shift in incentives promotes cooperative behavior among individuals.

Our findings shed light on the impact of strategy-dependent dynamics of vaccine behavior, revealing variations in final epidemic size and vaccination coverage. This has important implications for various issues, including vaccine reliability, vaccination cost, vaccine dilemma, benefit assessment, refusal rates, free riding, and disease incidence. Our model provides a valuable framework for quantifying the cost–benefit analysis and dilemmas inherent in vaccination games. It becomes evident that increasing vaccine efficacy and benefits while simultaneously reducing vaccination costs leads to an increase in vaccination coverage, ultimately reducing the final epidemic size.

Further, our study highlights that their cooperation or defection strategies influence individuals’ strategy-updating processes. We observed differences between traditional IB-RA and SB-RA strategy-updating processes, which have implications for understanding the dynamics of strategy adoption. Through our analysis, we identified social dilemma situations for each scenario, employing the concepts of FES and FOV deficiency to determine strategies that minimize such dilemmas. This approach enhances our understanding of how different combinations of cost–benefit analyses can improve the final epidemic size, taking into account factors like vaccination reliability and strategies. Despite the advantages of our approach, there have been limited efforts to apply this theoretical framework to the evolution of cognitive factors that influence strategy selection, including beliefs, ideas, behaviors, social influence, persuasion, and the associated costs and benefits. Additionally, there is potential to expand our research to incorporate an optimal control analysis into the existing general model, utilizing the mixed-strategy approach introduced in this study. Also, it is worth noting that these models find practical applications in the context of vaccine strategies for livestock farming [40] to optimize vaccination programs and mitigate the spread of infectious diseases among animals, which is crucial for maintaining the health and productivity of livestock populations.

## Figures and Tables

**Table 1:** Payoff structure for the fractions of four individuals.

Strategy	Healthy	Infected
Vaccinated	HV (Healthy and vaccinators)	IV (Infected and vaccinators)
	$x\;exp[-\left(1-\eta \right)R_{0}R\left(x,\infty \right)]$	$x(1-exp\left[-\left(1-\eta \right)R_{0}R\left(x,\infty \right)\right])$
Non-vaccinated	SFR (Successful Free-Rider)	FFR (Failed Free-Rider)
	$\left(1-x)exp[-R_{0}R\left(x,\infty \right)\right]$	$\left(1-x)(1-exp\left[-R_{0}R\left(x,\infty \right)\right]\right)$

**Table 2:** Cost–benefit payoff matrix of vaccination game (without normalization).

SFR	HV	FFR	IV
B	$B-C_{v}$	$-C_{d}$	$-C_{v}-C_{d}$

**Table 3:** Cost–benefit payoff matrix after using additive properties (without normalization).

SFR	HV	FFR	IV
0	$-C_{v}$	$-C_{d}-B$	$-C_{v}-C_{d}-B$

**Table 4:** Normalized cost–benefit payoff matrix.

SFR	HV	FFR	IV
0	$-C_{v}/(C_{d}+B)$	−1	$-C_{v}/(C_{d}+B)-1$

**Table 5:** Conventional vaccination game with normalization.

	SFR (Healthy)	HV (Healthy)	FFR (Infected)	IV (Infected)
Payoff	0	$-C_{r}$	−1	$-C_{r}-1$
Payoff gap	$C_{r}$			
		$1-C_{r}$		
			$C_{r}$	

**Table 6:** Proposed cost–benefit vaccination game with normalization.

	SFR (Healthy)	HV (Healthy)	FFR (Infected)	IV (Infected)
Payoff	0	$-\frac{C_{v}}{C_{d}+B}$	−1	$-\frac{C_{v}}{C_{d}+B}-1$
Payoff gap	$\frac{C_{v}}{C_{d}+B}$			
		$1-\frac{C_{v}}{C_{d}+B}$		
			$\frac{C_{v}}{C_{d}+B}$	

**Table 7:** Transition probability of *IB-RA.*

Transition Probability
Original form of PW-Fermi considering each agent; $Pr\left(s_{i}\leftarrow s_{j}\right)=\frac{1}{1+\exp[-(\pi_{j}-\pi_{i})/\kappa ]}$
$Pr\left(SFR\leftarrow HV\right)=\frac{1}{1+\exp[-(-C_{v}/(C_{d}+B)-0)/\kappa ]}$ $Pr\left(FFR\leftarrow HV\right)=\frac{1}{1+\exp[-(-C_{v}/(C_{d}+B)+1)/\kappa ]}$ $Pr\left(SFR\leftarrow IV\right)=\frac{1}{1+\exp[-(-C_{v}/(C_{d}+B)-1-0)/\kappa ]}$ $Pr\left(FFR\leftarrow IV\right)=\frac{1}{1+\exp[-(-C_{v}/(C_{d}+B)-1+1)/\kappa ]}$ $Pr\left(HV\leftarrow SRF\right)=\frac{1}{1+\exp[-(0+C_{v}/(C_{d}+B))/\kappa ]}$ $Pr\left(HV\leftarrow FFR\right)=\frac{1}{1+\exp[-(-{1+C}_{v}/(C_{d}+B))/\kappa ]}$ $Pr\left(IV\leftarrow SFR\right)=\frac{1}{1+\exp[-(0+C_{v}/(C_{d}+B)+1)/\kappa ]}$ $Pr\left(IV\leftarrow FFR\right)=\frac{1}{1+\exp[-(1+C_{v}/(C_{d}+B)+1)/\kappa ]}$

**Table 8:** Transition probability of *SB-RA.*

	Transition Probability
	Fermi pairwise rules for SB-RA, $Pr\left(s_{i}\leftarrow {<\pi }_{j}>\right)=\frac{1}{1+\exp[-({<\pi }_{j}>-S_{i})/\kappa ]}$
	$Pr\left(HV\leftarrow NV\right)=\frac{1}{1+\exp[-(\pi_{D}+C_{v}/(C_{d}+B))/\kappa ]}$ $Pr\left(IV\leftarrow NV\right)=\frac{1}{1+\exp[-(\pi_{D}+C_{v}/(C_{d}+B)+1)/\kappa ]}$ $Pr\left(SFR\leftarrow V\right)=\frac{1}{1+\exp[-(\pi_{C}-0)/\kappa ]}$ $Pr\left(FFR\leftarrow V\right)=\frac{1}{1+\exp[-(\pi_{C}+1)/\kappa ]}$

## Data Availability

No data was used for the research described in the article.

## References

[1] Ozkan-Canbolat E., Beraha A., Bas A. (2016). Application of Evolutionary Game Theory to Strategic Innovation. Procedia Soc. Behav. Sci..

[2] Feng Z. (2007). Final and peak epidemic sizes for SEIR models with quarantine and isolation. Math. Biosci. Eng..

[3] Safi M.A., Imran M., Gumel A.B. (2012). Threshold dynamics of a non-autonomous SEIRS model with quarantine and isolation. Theory Biosci..

[4] Chang S.L., Piraveenan M., Pattison P., Prokopenko M. (2020). Game theoretic modelling of infectious disease dynamics and intervention methods: A review. J. Biol. Dyn..

[5] Anderson R.M., May R.M. (1985). Vaccination and herd immunity to infectious diseases. Nature.

[6] Struchiner C.J., Halloran M.E., Spielman A. (1989). Modeling malaria vaccines I: New uses for old ideas. Math. Biosci..

[7] Galvani A.P., Reluga T.C., Chapman G.B. (2007). Long-standing influenza vaccination policy is in accord with individual self-interest but not with the utilitarian optimum. Proc. Natl. Acad. Sci. USA.

[8] Anderson R.M., May R.M. (1992). Infectious Diseases of Humans.

[9] Coburn B.J., Wagner B.G., Blower S. (2009). Modeling influenza epidemics and pandemics: Insights into the future of swine flu (H1N1). BMC Med..

[10] Longini I.M., Halloran J.M.E., Nizam A., Yang Y. (2004). Containing pandemic influenza with antiviral agents. Am. J. Epidemiol..

[11] Viboud C., Boelle P.Y., Carrat F., Valleron A.J., Flahault A. (2000). Prediction of the spread of influenza epidemics by the method of analogues. Am. J. Epidemiol..

[12] Viboud C., Boelle P.Y., Pakdaman K., Carrat F., Valleron A.J., Flahault A. (2004). Influenza epidemics in the United States, France, and Australia, 1972–1997. Emerg. Infect. Dis..

[13] Kuga K., Tanimoto J. (2018). Which is more effective for suppressing an infectious disease: Imperfect vaccination or defense against contagion?. J. Stat. Mech. Theory Exp..

[14] Kabir K.M.A., Kuga K., Tanimoto J. (2019). Effect of Information spreading to suppress the disease contagion on the epidemic vaccination game. Chaos Solitons Fractals.

[15] Kermack W.O., McKendrick A.G. (1927). A contribution to the mathematical theory of epidemics. Proc. Roy. Soc. London. Ser. A.

[16] Anderson R.M., May R.M. (1979). Population Biology of Infectious Diseases: Part, I. Nature.

[17] d’Onofrio A., Manfredi P., Salinelli E. (2007). Vaccinating behaviour, information, and the dynamics of SIR vaccine preventable diseases. Theor Popul Biol..

[18] Cardillo A., Reyes-Suárez C., Naranjo F., Gómez-Gardeñes J. (2013). Evolutionary vaccination dilemma in complex networks. Phy. Rev. E.

[19] Pennisi E. (2009). On the origin of cooperation. Science.

[20] Hofmann L.M., Chakraborty N., Sycara K. The evolution of cooperation in self-interested agent societies: A critical study. Proceedings of the 10th International Conference on Autonomous Agents and Multiagent Systems.

[21] Bauch C.T., Bhattacharyya S. (2012). Evolutionary game theory and social learning can determine how vaccine scares unfold. PLoS Comput. Biol..

[22] Chen X., Fu F. (2018). Social learning of prescribing behavior can promote population optimum antibiotic use. Front. Phys..

[23] Zuo C., Ling Y., Zhu F., Ma X., Xiang G. (2023). Exploring epidemic voluntary vaccinating behavior based on information-driven decisions and benefit-cost analysis. Appl. Math Comput..

[24] Lim W., Zhang P. (2020). Herd immunity and a vaccination game: An experimental study. PLoS ONE.

[25] Altwaijri N., Abualait T., Aljumaan M., Albaradie R., Arain Z., Bashir S. (2022). Defense mechanism responses to COVID-19. Peer J..

[26] dOnofrio A., Manfredi P., Poletti P. (2011). The impact of vaccine side effects on the natural history of immunization programmes: An imitation-game approach. J. Theor. Biol..

[27] Fu F., Rosenbloom D.I., Wang L., Nowak M.A. (2011). Imitation dynamics of vaccination behaviour on social networks. P. Roy. Soc. B-Biol. Sci..

[28] Kabir K.M.A., Jusup M., Tanimoto J. (2019). Behavioral incentives in a vaccination-dilemma setting with optional treatment. Phys. Rev. E..

[29] Kabir K.M.A. (2021). How evolutionary game could solve the human vaccine dilemma. Chaos Solitons Fractals.

[30] Butt A.I.K., Ahmad W., Rafiq M., Ahmad N., Imran M. (2023). Computationally efficient optimal control analysis for the mathematical model of Coronavirus pandemic. Expert Syst. Appl..

[31] Hanif A., Butt A.I.K., Ahmad W. (2023). Numerical approach to solve Caputo-Fabrizio-fractional model of corona pandemic with optimal control design and analysis. Mathematical Methods in Applied Sciences.

[32] Gaff H., Schaefer E. (2009). Optimal control applied to vaccination and treatment strategies for various epidemiological models. Math. Biosci. Eng..

[33] Kabir K.M.A. (2023). Impact of human cooperation on vaccination behaviors. Heliyon.

[34] Caley P., Philp D.J., McCracken K. (2008). Quantifying social distancing arising from pandemic influenza. J. R. Soc. Interface.

[35] Zaman G., Kang Y.H., Cho G., Jung I.H. (2017). Optimal strategy of vaccination and treatment in an 415 SIR epidemic model. Math. Comput. Simul..

[36] Perc M., Szolnoki A. (2010). Coevolutionary games–a mini review. Biosystems.

[37] Howard J., Huang A., Li Z., Tufekci Z., Zdimal V., Van Der Westhuizen H.M., von Delft A., Price A., Fridman L., Tang L.-H. (2021). An evidence review of face masks against COVID-19. Proc. Natl. Acad. Sci. USA.

[38] Bauch C.T., Earn D.J. (2004). Vaccination and the theory of games. Proc. Natl. Acad. Sci. USA.

[39] Arefin M.R., Kabir K.M.A., Jusup M., Ito H., Tanimoto J. (2020). Social efficiency deficit deciphers social dilemmas. Sci. Rep..

[40] Pandey S., Kalwa U., Kong T., Guo B., Gauger P.C., Peters D.J., Yoon K.-J. (2021). Behavioral Monitoring Tool for Pig Farmers: Ear Tag Sensors, Machine Intelligence, and Technology Adoption Roadmap. Animals.

